# Severe leptospirosis infection in a non-epidemic area

**DOI:** 10.1016/j.idcr.2021.e01345

**Published:** 2021-11-20

**Authors:** Rioto Suzuki, Mari Terayama, Minoru Tanda

**Affiliations:** Iwate Prefecture Advanced Critical Care and Emergency Center, Iwate Medical University, Japan

**Keywords:** Leptospirosis, Pulmonary hemorrhage, Acute kidney injury, Weil’s disease

## Abstract

A 39-year-old man visited our hospital with lower leg pain and fever. We suspected sepsis because of an infectious disease. He was hospitalized, and treatment was initiated. After admission, we received information that mice were present in his living environment. Moreover, we considered leptospirosis in the differential diagnosis and started the administration of ceftriaxone and minocycline. On the 10th day after admission, after examination by the National Institute on Infectious Diseases, we diagnosed him with leptospirosis. The patient was transferred to the hospital for rehabilitation on day 23 after admission. It is important to consider leptospirosis even in non-epidemic area.

## Introduction

Leptospirosis is a zoonotic bacterial infection that in caused by the spirochete *Leptospira* spp. Severe leptospirosis is called Weil’s disease. Leptospirosis was designated as a Class-4 infectious disease by the Infectious Diseases Control Law in 2003, and 494 outbreaks were reported in Japan from 2003 to 2018. In the Iwate prefecture, there were no reports of outbreaks except for one patient who was thought to have been infected during a trip to the Okinawa prefecture in 2013. We report the case of a patient with severe leptospirosis who was thought to have been infected in the Iwate prefecture and had pulmonary hemorrhage, thrombocytopenia, and acute kidney injury.

## Case report

A 39-year-old man became aware that he was feeling fatigue the end of July 2020; he gradually also experienced lower leg pain and headache. He had diarrhea and vomiting; therefore, he visited a local hospital. He was referred to our hospital from the local hospital on the next day. We urgently hospitalized him, suspecting an infectious disease. His medical history only included a gastric ulcer. He was engaged in forestry and smoked for about 10 pack-years. He had no history of domestic or international travel and no recent history of sexual intercourse.

On examination, he was conscious and alert. His vital signs included a heart rate of 114 beats/min, blood pressure of 109/79 mmHg, and body temperature of 39.4 ℃. His blood oxygen saturation was 96%; however, bloody sputum was present. Chest auscultation revealed no rales. He complained of headache and severe pain in the lower legs. The clinical examination was otherwise unremarkable.

The laboratory data on admission have been shown in Table 1. In the peripheral blood, the white blood cell count was increased, predominantly, the neutrophils; his C-reactive protein level was also. Thrombocytopenia was present. His transaminase level was elevated; however, there was no hyperbilirubinemia. There was an increase in the urea nitrogen and serum creatinine levels. Granular casts and epithelial casts appeared in the urine, suggesting renal injury.

**Table 1:** Laboratory finding on admission.

Hematology		Biochemistry and serology	Coagulation		
WBC (/μL)	9204	TP (g/dL)	6.6	PT (sec)	11.8
Neut (%)	92.0	Alb (g/dL)	3.1	PT-INR	1.00
Lym (%)	6.0	T-bil (mg/dL)	0.6	APTT (sec)	40.6
Mo (%)	2.0	AST (U/L)	52	Fbg (mg/dL)	763
Eos (%)	0.0	ALT (U/L)	99	D-D (μg/mL)	0.8
Baso (%)	0.0	γ-GTP (U/L)	66		
RBC (×10^6^/μL)	405	LDH (U/L)	224	Urinalysis	
Hb (g/dL)	12.7	CK (U/L)	410	Protein (g/gCr)	0.30
Ht (%)	35.9	BUN (mg/dL)	44.8	Glucose	–
Plt (×10^4^/μL)	2.3	Cre (mg/dL)	3.13	RBC (/HPF)	5–9
		Na (mmol/L)	130	WBC (/HPF)	50–99
		K (mmol/L)	3.9	Hyalyne cast	+
		Cl (mmol/L)	93	Granular cast	+
		Ca (mg/dL)	7.7	Epithelial cast	+
		CRP (mg/dL)	27.7		

WBC: white blood cell, Neut: neutrophil, Lym: lymphocyte, Mo: monocyte, Eos: eosinophil, Baso: basophil, RBC: red blood cell, Hb: hemoglobin, Ht: hematocrit, Plt: platelet, TP: total protein, Alb: albumin, T-bil: total birilubin, AST: aspartate aminotransferase, ALT: alanine aminotransferase, γ-GTP: gamma-glutamyl transpeptitase, LDH: lactate dehydrogenase, CK: creatinine kinase, BUN: blood urea nitrogen, Cre: creatinine, CRP: C-reactive protein, PT: prothrombin time, PT-INR: prothrombin time-international normalized ratio, APTT: activated partial thromboplastin time, Fbg: fibrinogen, D-D: d-dimer, HPF: high power field.

**Fig. 1:**
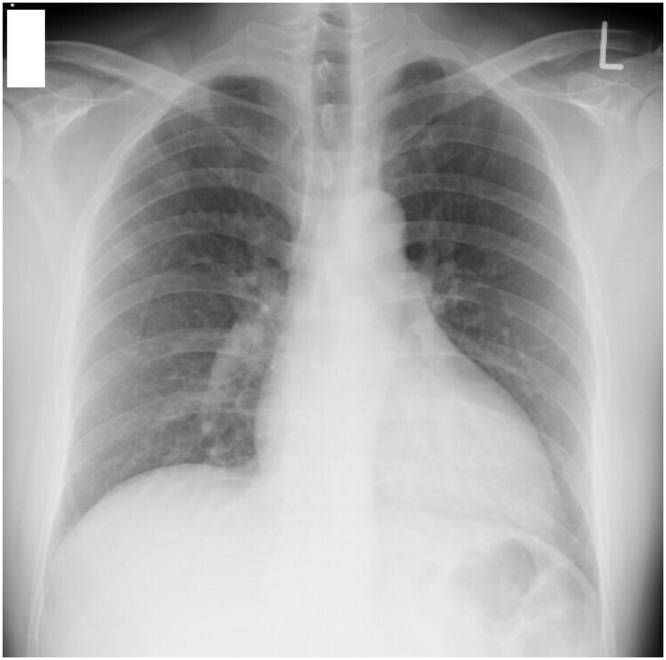
Chest radiograph on admission.

**Fig. 2:**
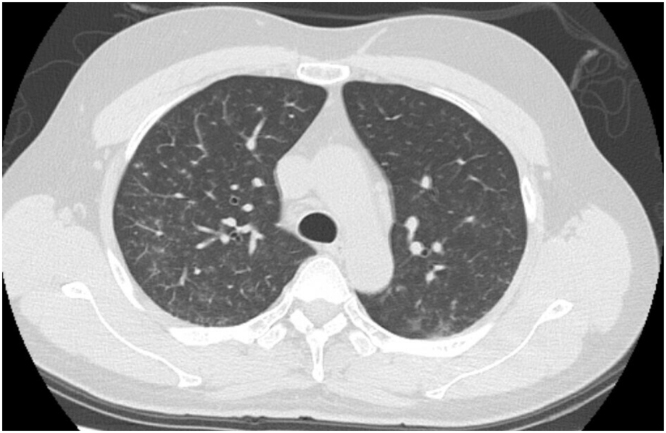
Chest computed tomography on admission.

The imaging finding on admission have been shown in Figure. Chest radiographs did not reveal infiltration shadows (Fig. 1). Computed tomography showed central lobular shadows and pale ground-grass shadows in both the lungs (Fig. 2), suggesting alveolar hemorrhage along with bloody sputum symptoms.

**Fig. 3:**
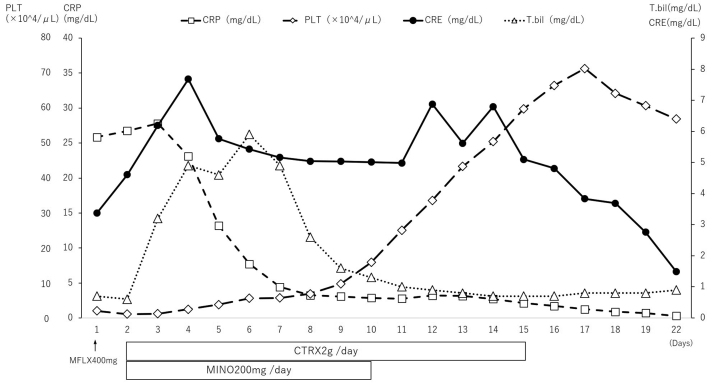
Clinical course of the patient. CRE: creatinine, CRP: C-reactive protein, CTRX: ceftriaxone, MFLX: moxifloxacin, MINO: minocycline, PLT: platelets, T.bil: total bilirubin.

The patient’s clinical course has been described in Fig. 3. After admission, we administered 400 mg of moxifloxacin. On day 2 of admission, we received information that there were mice in the patient’s living environment. Ceftriaxone at 2 g/d and minocycline at 200 mg/d were started, because we considered leptospirosis and rickettsia infection in the differential diagnosis. Immunoglobulin was also administered for 3 d for severe infection. Oxygen therapy was started with a high flow nasal cannula because of respiratory failure. On day 3 of admission, hyperemia of the bulbar conjunctiva and direct bilirubin-dominant bilirubin elevation appeared. Hemodialysis was started because of exacerbation of acute kidney injury by day 4 of admission. Leptospirosis was highly likely owing to the presence of symptoms, such as thrombocytopenia, acute kidney injury, liver injury, and hyperbilirubinemia. Thus, we requested the National Institute of Infectious Disease to perform tests using blood and urine samples. On day 10 of admission, the National Institute of Infectious Disease provided the results; the PCR of the urine samples showed positive leptospiral DNA; thus, a diagnosis of leptospirosis was established. Antimicrobial administration and hemodialysis were completed on day 15 of admission to reduce the inflammation and treat acute kidney injury. Thrombocytopenia also started subsiding. Oxygen therapy was completed on day 16 of admission to treat respiratory failure. He was transferred to a local hospital on day 23 of admission for rehabilitation. Thereafter, the results of a microscopic agglutination test (MAT) performed on blood samples on day 22 of admission were received (Table 2). The paired sera on day 4 and day 22 were significantly increased in the titers of the two types of antibodies (*Leptospira interrogans* serovar Autumnalis and *Leptospira interrogans* serovar Rachmati).

**Table 2:** Result of microscopic aggluntination test.

Test piece	Antibody titer	
	On day 4 of admission	On day 22 of admission
*Leptospira borgpetersenii* serovar Poi	<50	400
*Leptospira interrogans* serovar Autumnalis	<50	800
*Leptospira interrogans* serovar Hebdomadis	<50	200
*Leptospira interrogans* serovar Rachmati	<50	800

## Discussion

*Leptospira* spp. colonizes the renal tubules of many mammals, mainly rodents, and is excreted in the urine. Humans are infected percutaneously or transmucosally via direct contact with the urine of leptospiral carries and contact with soil contaminated with such urine [1]. Leptospirosis is prevalent worldwide, mainly in tropical and subtropical regions, and many cases have been reported in Southeast Asia and Central and South America. Cases of imported infection have also been reported. Sporadic outbreaks are seen nationwide in Japan; however many cases have been reported, especially in the Okinawa prefecture. Leptospirosis outbreak occasionally occurs in cases of flood due to a typhoon or heavy rain. In the present case, a heavy rain or flood warning was issued in the residential area about 2–3 wk before the patient started experiencing the symptoms, and the river was flooded. In this case, we received information that there were mice in the patient’s living environment, and it was considered that the cause of the infection was direct contact with manure. In addition to contact with the mice, it is considered that the course of this infection is also affected by the infection route.

Leptospirosis has an incubation period of about 5–14 days, and presents with various clinical symptoms. Mild leptospirosis is with only subclinical infection or cold symptoms. Severe leptospirosis is called Weil’s disease with jaundice, bleeding, and renal disorder as the three main symptoms [2]. In addition, lower leg pain that was reported by our patient is considered a characteristic symptom [3]. In this case, there was no jaundice at the time of admission; however, on day 3 of admission, bilirubin was directly predominantly elevated, and jaundice appeared. In addition, alveolar hemorrhage, acute respiratory distress syndrome, and pulmonary edema have been reported as lung lesions associated with leptospirosis [4], [5]. Serious pulmonary complications were observed in 2.2% of the 321 cases of leptospirosis, and it was reported that 4 out of 5 patients died because of alveolar hemorrhage [4]. It is considered that the lung lesion in this patient suggested alveolar hemorrhage. We performed oxygen therapy with a high flow nasal cannula and achieved good treatment outcome. However, as per a previous report the introduction of veno-venous extra-corporeal membrane oxygenation (VV-ECMO) saved patient’s lives in case of severe disease [6], and it is necessary to take measures as per the patient’s respiratory function. Serum diagnosis using MAT is useful for diagnosing leptospirosis [1]. In this case, antibody titers of the two types significantly increased (*Leptospira interrogans* serovar Autumnalis and *Leptospira interrogans* serovar Rachmati). We should consider the cross-reactivity for the other two types (*Leptospira borgpetersenii* serovar Poi and *Leptospira interrogans* serovar Hebdomadis). The PCR method is also useful; however, it requires samples to be collected in the acute phase. In this case, leptospiral DNA was not detected in blood samples; however, leptospiral DNA was detected in the urine samples 4 d after admission. In the case of the PCR method, the results were consistent with the report [7] that long-term detectable urine samples were suitable for diagnosis. The result of MAT, that was received later showed a significant increase in the antibody titer, and the patient was diagnosed with leptospirosis. For treatment, doxycycline is recommended for mild to moderate cases, and penicillin g is recommended for severe cases [8]. Ceftriaxone used in this case reportedly exerts the same therapeutic effect as penicillin g [9]. Leptospirosis in known to cause a Jarisch-Herxheimer reaction after the administration of antibacterial agents [10]; however, no such reaction was observed in our patient.

We treated a patient with severe leptospirosis, although leptospirosis is a rare disease in non-epidemic areas and is difficult to distinguish. However, we reiterate that it is important to establish a diagnosis and perform early intervention by conducting a detailed medical history and living environment interview based on the symptoms.

## Funding

There is no funding source for research.

## Author contribution

(1) The conception and design of the study, or acquisition of data, or analysis and interpretation of data, (2) drafting the article or revising it critically for important intellectual content, (3) final approval of the version to be submitted. All authors meet the above ICMJE authorship criteria. Mari Terayama, Minoru Tanda: The authors have contributed to this report in terms of joint medical treatment.

## Consent

Written informed consent was obtained from the patient for publication of this case report and accompanying images. A copy of the written consent is available for review by the Editor-in-Chief of this journal on request.

## Ethical approval

This report has been approved by the Ethics Committee.

## Conflict of Interest

The authors state that they have no Conflict of Interest (COI).
